# Click evoked otoacoustic emissions in occupational exposure to lead, concentrations of selected essential elements and markers of oxidative stress

**DOI:** 10.1007/s00405-024-08675-0

**Published:** 2024-05-02

**Authors:** Marta Wąsik, Grażyna Lisowska, Michał Słota, Katarzyna Miśkiewicz-Orczyk, Aleksandra Kasperczyk, Francesco Bellanti, Michał Dobrakowski, Rafał Jakub Bułdak, Sławomir Kasperczyk

**Affiliations:** 1 Department of Clinical Biochemistry and Laboratory Diagnostics, Institute of Medicine, Opole University, Oleska 48, 45-052 Opole, Poland; 2 Department of Otorhinolaryngology and Laryngological Oncology, Medical University of Silesia in Katowice, Skłodowskiej-Curie 10, 41-840 Zabrze, Poland; 3 ARKOP Sp. z o.o., Kolejowa 34a, 32-332 Bukowno, Poland; 4 Department of Biochemistry, Faculty of Medical Sciences in Zabrze, Medical University of Silesia in Katowice, Jordana 19, 41-808 Zabrze, Poland; 5 Department of Medical and Surgical Sciences, University of Foggia, Viale Pinto 1, 71122 Foggia, Italy

**Keywords:** CEOAE, Hearing assessment, Blood tests, Lead, Cadmium, Calcium, Zinc, Malondialdehyde, Lipofuscin

## Abstract

**Purpose:**

This study focused on the selected markers of oxidative stress, impact of elevated lead levels on long-term hearing quality. We investigated whether the presence of certain essential minerals might provide protection to the auditory system against the effects of lead (and cadmium) compounds.

**Methods:**

The research group included 280 male employees of the zinc and lead smelter, which was divided into: L-Pb—low blood lead concentration (PbB) subgroup, H-Pb—high PbB subgroup. Hearing tests were performed using the click evoked otoacoustic emission (CEOAE).

**Results:**

Zinc protoporphyrin level was significantly higher in the H-Pb subgroup by 68%. Cd concentration was significantly higher in H-Pb by 33%. The Ca concentration was significantly lower in the H-Pb by − 2%. Selected oxidative stress markers concentration were significantly higher in the H-Pb group: malondialdehyde (MDA) by 4%, and lipofuscin (LPS) by 9%. In the CEOAE results showed statistically significant differences between the L-Pb and H-Pb subgroups. Larger negative changes in otoemission amplitude were observed in H-Pb subgroup. All otoemission results showed a statistically significant negative correlation with age, time of work, MDA concentration, and with PbB. Selected CEOAE parameters showed a significant negative correlation with cadmium blood concentration (CdB), and a positive correlation with Ca and Zn.

**Conclusion:**

Elevated blood lead content in occupational exposure is associated with an increase in MDA and LPS concentration, which negatively correlates with CEOAE parameters. This suggests an important role of oxidative stress in the long-term deterioration of hearing.

## Introduction

Lead is one of the most commonly used metals, and lead poisoning is the most common heavy metal poisoning [1]. Lead compounds causes many negative effects on the human body: affects the central and peripheral nervous system (encephalopathy, neuropathy, demyelination and nerve conduction disorders) [2], affects the hematopoietic and circulatory system (hemoglobin synthesis disorders, anemia, hypertension) [3, 4], can induce cardiovascular disorders due to immune-modulation, oxidative, and inflammatory mechanisms [5], has a negative effect on the renal system (nephropathy) [3], induces cellular oxidative stress (excessive amount of reactive oxygen species (ROS), dysfunction of antioxidant enzymatic and non-enzymatic systems) [5]. It has been shown that exposure to lead has a negative effect on human auditory system [6]. This is due to the negative impact of lead on the central nervous system and distribution of nerve conduction velocities and peripheral nerve conduction velocity [2]. Molecular ototoxic factors pf Pb include the negative impact of oxidative stress, modification of the properties of cell membranes, disorders of signaling pathways causing degeneration and inhibition of neuronal growth, and disruption of the release of neurotransmitters [7].

The aim of this study was to compare a group of employees with a low level of lead with a group of employees with a high level of lead in terms of selected markers of oxidative stress and the proper functioning of the sense of hearing. Long-term exposure resulting in elevated blood lead levels (and co-occurring cadmium) was investigated. In addition, it was analyzed whether the content of selected minerals may have a protective effect on the hearing organ when exposed to lead (and cadmium) compounds.

## Material and methods

### Study population

The research group included male employees of the zinc and lead smelter in Miasteczko Śląskie (Poland) and consisted of 280 men worked in various positions in the steelworks and were exposed to lead and cadmium compounds. The following data were obtained from the personal questionnaire: age, body weight, height, time of work without exposure to heavy metals, time of work in exposure to lead and cadmium, and smoking behavior. In addition, a medical history was taken. The study population was divided into two subgroups based on the median PbB value (= 35 µg/dl): L-Pb: low PbB subgroup, PbB < 35 µg/dl, n = 140, H-Pb: high PbB subgroup, PbB ≥ 35 µg/dl, n = 140.

### Audiometric testing

All subjects underwent otoscopy verification. If cerumen prevented proper examination or the eardrum looked abnormal, the patient was excluded from the study and consulted an otolaryngologist.

Hearing tests were performed using the click evoked otoacoustic emission (CEOAE). The test was performed with a Capella equipment (Madsen Electronics, Denmark). Lightweight probes fitted with an appropriately sized disposable ear tip were inserted into the subject's ears. The sealing of the probe insertion was checked. A standard default setup was used: the test stimulus was an ipsilateral click 80 µs duration of 80 dB sound pressure level (SPL). Both ears were tested separately, analyzed separately and together. CEOAE was analyzed for the frequency bands of 1, 1.5, 2, 3, 4 kHz. For further analysis the mean values were calculated over the entire range from 1 to 4 kHz, and from 2 to 4 kHz range (to eliminate patients with conductive hearing loss (independent of the factors considered in this paper). The decision criterion for the presence of CEOAE was a reproducibility ≥ 50%, and with a signal-to-noise ratio ≥ 3 dB [8]. Only patients with measurable otoemissions in at least one ear were included in further analysis. The following parameters were recorded: reproducibility (Rep, [%]), amplitude (Amp, [%]) and signal-to-noise ratio (SNR, [dB]), for the right (R) and left (L) ear separately. The results from both ears or from one ear with the presence of otoemission (if the otoemission was not binaural) were considered as the bilateral mean value.

### Laboratory procedures

Whole blood was collected and laboratory tests were performed based on the methodology described in detail in our previous article [9]. The biochemical test was performed: blood lead concentration (PbB), blood cadmium concentration (CdB), blood concentration of zinc protoporphyrin (ZPP). The assessments of the serum concentrations of selected essential elements were performed: iron (Fe), calcium (Ca), magnesium (Mg), and zinc (Zn). The concentration of selected markers of oxidative stress was examined: malondialdehyde (MDA) and lipofuscin (LPS).

### Statistical analysis

The statistical analysis was performed using the STATISTICA 13 software program (StatSoft). Descriptive statistics were presented as mean ± standard deviation (SD) for variables with a normal distribution. The initial assessment of normality of distribution for each variable was conducted using the Shapiro–Wilk test, while the homogeneity of variances was confirmed through Levene’s test. Statistical analyses encompassed Student’s t-test, Mann–Whitney U test, and chi-squared test, as appropriate. Spearman's rank correlation analysis was performed to determine possible relationships between lead concentrations and demographic variables, as well as relationships between parameters values of otoacoustic emissions. Statistical significance was defined as a probability of *p* ≤ 0.05.

### Study scheme

**Fig. 1:**
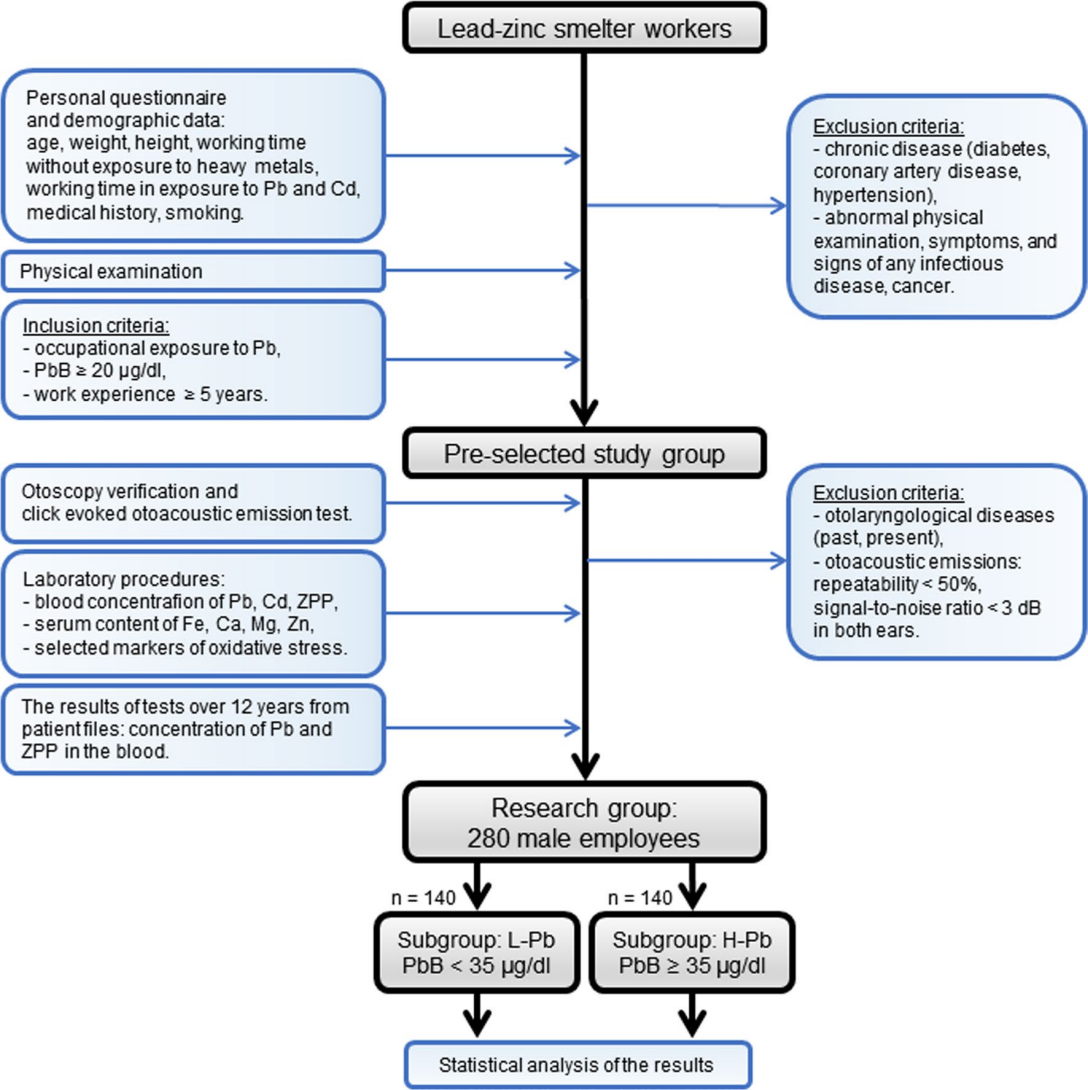
Flowchart of the study

The study design, tests sequence, inclusion and exclusion criteria are presented in the flowchart below (Fig. 1).

## Results

L-Pb and H-Pb subgroups were homogeneous: they did not differ significantly in mean age, sex, years of work non-exposed to lead, mean body weight, height and smoking habit (Table 1). The subgroups differed in years of work in lead exposure: L-Pb = 12.40, H-Pb = 14.90, *p* = 0.020.

**Table 1:** Physical measurements and health habits

		Subgroup L-Pb PbB < 35 [µg/dl] n = 140		Subgroup H-Pb PbB ≥ 35 [µg/dl] n = 140		Variation [%]	*p* value^*^
		Mean	SD	Mean	SD		
Age [years]		40.40	9.90	42.20	8.49	4%	**0.104**
Years of work in exposure to Pb [years]		12.40	9.32	14.90	8.22	20%	*0.020*
Years of work without Pb exposure [years]		28.00	9.57	27.30	7.25	− 2%	**0.518**
Body weight [kg]		85.20	12.50	87.50	14.50	3%	**0.231**
Height [cm]		177.00	6.28	177.00	5.98	0%	**0.727**
Body mass index (BMI)		27.00	3.60	27.90	4.01	3%	**0.143**
Smoking:	Yes/No [%]	21%		32%			**0.084**
	In the past [years]	12.73	7.61	13.56	7.52	7%	**0.568**
	Currently [amount]	12.62	6.79	11.27	5.35	− 11%	**0.449**

^*^Statistical significance *p* ≤ 0.05, italic font; bold font—no statistical significance

The subgroups differed in PbP mean concentration (dividing criterion): in L-Pb was PbB = 25.58 µg/dl, in H-Pb was PbB = 41.08 µg/dl, *p* < 0.001 (Table 2). ZPP content was significantly higher in the H-Pb subgroup by 68% (lead-dependent parameter), *p* < 0.001. CdB level was significantly higher in H-Pb by 33%, *p* < 0.001. The calcium concentration was significantly lower in the H-Pb subgroup by 2%, *p* = 0.037. Other tested minerals (Fe, Mg, Zn) showed no statistical difference between the subgroups. Selected oxidative stress markers were significantly higher in the H-Pb group: MDA concentration by 4%, *p* = 0.041, and LPS concentration by 9%, *p* = 0.040.

**Table 2:** Biochemical blood test results

	Subgroup L-Pb PbB < 35 [µg/dl] n = 140		Subgroup H-Pb PbB ≥ 35 [µg/dl] n = 140		Variation [%]	*p* value^*^
	Mean	SD	Mean	SD		
PbB [µg/dl]	25.58	7.94	41.08	4.40	61%	< *0.001*
ZPP [µg/g Hb]	3.94	1.94	6.62	2.76	68%	< *0.001*
CdB [µg/l]	2.00	1.76	2.66	2.10	33%	< *0.001*
Fe [µg/dl]	21.60	8.13	22.90	9.02	6%	**0.605**
Ca [mmol/l]	2.43	0.22	2.39	0.24	− 2%	*0.037*
Mg [mmol/l]	0.82	0.14	0.81	0.13	− 1%	**0.798**
Zn [mmol/l]	13.90	4.17	14.00	5.61	1%	**0.897**
MDA [µmol/l]	2.82	0.97	2.93	0.71	4%	*0.041*
LPS [RF/g Hb]	575.00	188.00	625.00	200.00	9%	*0.040*

^*^Statistical significance *p* ≤ 0.05, italic font; bold font—no statistical significance

The click evoked otoacoustic emission (CEOAE) results, for the right ear (R), the left ear (L), and the means for both ears combined (binaural), are shown in the Table 3. Statistically significant differences in the CEOAE results between L-Pb and H-Pb were demonstrated for the R ear for all tested parameters. Significant differences concerned both for the mean frequencies of 1–4 Hz and 2–4 Hz. For the L ear, significant differences between the subgroups were observed only in the Rep and SNR parameters, for the mean frequency of 1–4 Hz. In the mean results from both ears, significant differences between L-Pb and H-Pb were obtained in almost all tested parameters. In addition, it was observed that in the H-Pb subgroup the Amp 2–4 Hz parameter showed negative values (both for the R, L ears and binaural).

**Table 3:** Click evoked otoacoustic emission (CEOAE) results, for right ear (R), left ear (L) and both ears combined

	Click evoked otoacoustic emission parameters^a^		Subgroup L-Pb PbB < 35 [µg/dl] n = 140		Subgroup H-Pb PbB ≥ 35 [µg/dl] n = 140		Variation [%]	*p* value^*^
			Mean	SD	Mean	SD		
Right ear	1–4 kHz	Rep	87.0	15.6	80.8	21.3	− 7%	*0.007*
		Amp	13.5	4.77	12.0	5.54	− 11%	*0.016*
		SNR	14.2	5.26	12.2	5.73	− 14%	*0.003*
	2–4 kHz	Rep	68.5	28.2	58.4	30.6	− 15%	*0.006*
		Amp	1.41	6.54	-0.58	5.76		*0.009*
		SNR	11.0	6.37	8.62	5.70	− 21%	*0.002*
Left ear	1–4 kHz	Rep	85.1	16.1	80.5	19.7	− 5%	*0.042*
		Amp	12.6	4.97	11.6	5.18	− 9%	**0.088**
		SNR	13.3	5.11	11.8	5.21	− 11%	*0.024*
	2–4 kHz	Rep	65.5	30.1	58.5	30.0	− 11%	**0.063**
		Amp	0.07	5.87	-0.73	6.06		**0.286**
		SNR	9.61	5.74	8.41	5.84	− 12%	**0.099**
Binaural	1–4 kHz	Rep	85.7	14.1	80.5	18.7	− 6%	*0.010*
		Amp	12.9	4.63	11.7	4.88	− 9%	*0.033*
		SNR	13.6	4.84	11.9	4.95	− 12%	*0.005*
	2–4 kHz	Rep	65.6	27.2	58.6	28.6	− 11%	*0.037*
		Amp	0.40	5.88	− 0.67	5.62		**0.121**
		SNR	10.0	5.75	8.50	5.45	− 15%	*0.027*

^a^1-4—Mean values from 1 to 4 kHz (frequency: 1, 1.5, 2, 3, 4 kHz), 2–4—Mean values from 2 to 4 kHz (frequency: 2, 3, 4 kHz), Rep—Reproducibility [%], Amp—Amplitude [dB], SNR—Signal-to-Noise Ratio [dB]

^*^Statistical significance *p* ≤ 0.05, italic font; bold font—no statistical significance

The Spearman rank correlation results are presented in Tables 4 and 5. All otoemission results showed a statistically significant negative correlation with age, time of work in lead exposure and without exposure to this metal. Otoemission results showed a statistically significant negative correlation with PbB, in almost all otoemission parameters. The results of the otoemission test partially showed a statistically significant negative correlation with the ZPP concentration, mainly the following parameters for the average frequencies of 1–4 Hz: Amp (ear L, ear R and binaural), Rep and SNR (ear L and binaural). The CbB level showed a statistically significant negative correlation with the otoemission parameters of the L ear; moreover, the CdB showed a positive correlation with the 12-year average value of PbB and the 12-year average of ZPP value. The level of Ca showed a statistically significant positive correlation with the otoemission results of ear L, at the mean of frequency 1–4 and 2–4 in the Rep and SNR parameters. The Zn concentration showed a significant positive correlation with selected CEOAE parameters, mainly Rep and SNR in the R ear and binaurally, at frequencies of 2–4 Hz. The concentrations of Fe and Mg did not show statistically significant correlations (not included in the table). The content of MDA showed a statistically significant negative correlation with all tested otoemission parameters; MDA concentration also correlated with the 12-year average PbB value. The content of LPS showed a negative correlation only in the otoemission parameters of the ear L at the mean of frequency 1–4: Rep, Amp, SNR.

**Table 4:** Spearman rank order correlation: click evoked otoacoustic emission (CEOAE)

	Click evoked otoacoustic emission parameters^a^		Age [years]	Work in exposure to Pb [years]	Work without Pb exposure [years]	PbB [µg/dl]	ZPP [µg/g Hb]	CdB [µg/l]	Ca [mmol/l]	Zn [mmol/l]	MDA [µmol/l]	LPS [RF/g Hb]
Right ear	1–4	Rep	− *0.30*	*-0.18*	− *0.16*	− *0.15*					− *0.19*	
		Amp	− *0.22*	− *0.13*	− *0.17*	− *0.15*	− *0.12*				− *0.17*	
		SNR	− *0.29*	− *0.17*	− *0.16*	− *0.15*				*0.16*	− *0.20*	
	2–4	Rep	− *0.46*	− *0.28*	− *0.22*	− *0.20*	− *0.15*			*0.16*	− *0.20*	
		Amp	− *0.43*	− *0.25*	− *0.24*	− *0.16*	− *0.13*				− *0.20*	
		SNR	− *0.46*	− *0.27*	− *0.24*	− *0.19*	− *0.14*		*0.13*	*0.15*	− *0.19*	
Left ear	1–4	Rep	− *0.31*	− *0.19*	− *0.16*	− *0.14*	− *0.14*	− *0.14*	*0.14*		− *0.18*	− *0.13*
		Amp	− *0.25*	− *0.14*	− *0.18*	− *0.12*	− *0.14*	− *0.15*			− *0.14*	− *0.13*
		SNR	− *0.28*	− *0.17*	− *0.17*	− *0.13*	− *0.15*	− *0.15*	*0.15*		− *0.16*	− *0.14*
	2–4	Rep	− *0.47*	− *0.26*	− *0.25*	− *0.13*			*0.15*		− *0.22*	
		Amp	− *0.43*	− *0.25*	− *0.23*			− *0.18*		*0.17*	− *0.19*	
		SNR	− *0.43*	− *0.24*	− *0.25*	− *0.13*		− *0.15*	*0.14*		− *0.21*	
Binaural	1–4	Rep	− *0.32*	− *0.19*	− *0.16*	− *0.16*	− *0.14*				− *0.21*	
		Amp	− *0.24*	− *0.13*	− *0.18*	− *0.13*	− *0.14*				− *0.17*	
		SNR	− *0.30*	− *0.18*	− *0.16*	− *0.15*	− *0.13*				− *0.20*	
	2–4	Rep	− *0.49*	− *0.28*	− *0.24*	− *0.15*				*0.16*	− *0.22*	
		Amp	− *0.44*	− *0.25*	− *0.25*			− *0.15*		*0.14*	− *0.19*	
		SNR	− *0.47*	− *0.27*	− *0.25*	− *0.14*		− *0.14*		*0.15*	− *0.21*	

^a^1-4—Mean values from 1 to 4 kHz (frequency: 1, 1.5, 2, 3, 4 kHz), 2–4—Mean values from 2 to 4 kHz (frequency: 2, 3, 4 kHz), Rep—Reproducibility [%], Amp—Amplitude [dB], SNR—Signal-to-Noise Ratio [dB]

Statistical significance *p* ≤ 0.05, italic font

**Table 5:** Spearman rank order correlation: blood biochemistry

	Age [years]	Work in exposure to Pb [years]	Work without Pb exposure [years]	PbB [µg/dl]	ZPP [µg/g Hb]	CdB [µg/l]	Ca [mmol/l]	Zn [mmol/l]	MDA [µmol/l]	LPS [RF/g Hb]
PbB^a^ [µg/dl]		*0.17*			*0.68*	*0.30*			*0.15*	
ZPP^a^ [µg/g Hb]		*0.12*		*0.68*		*0.27*	*-0.12*			*0.14*
CdB [µg/l]	*0.14*	*0.15*		*0.30*	*0.27*					

^a^Twelve-year average value. Statistical significance *p* ≤ 0.05, italic font

## Discussion

Otoacoustic emissions tests are a good diagnostic tool used to assess the functioning of the cochlea [10]. This study showed statistically significant differences in the CEOAE results for the L-Pb and H-Pb subgroups, as well as a significant negative correlation between the results of CEOAE parameters and the PbB concentration. This proves the existence of a noticeable relationship between the PbB of workers exposed to lead compounds and the values of CEOAE: the higher the lead concentration in the blood, the worse the hearing measurement results were recorded.

The Rep parameter, as an indicator of reproducibility, showed small differences between the L-Pb and H-Pb subgroups: binaural Rep variation is -6% and -11% (1–4 and 2–4 kHz, respectively). The ototoxic effect of lead was the subject of studies by other authors, where a correlation was observed between the lead blood level and an increased hearing level, especially at high frequencies (3, 4, 6, 8 kHz) [11, 12].

In this study, larger negative changes in otoemission amplitude in the H-Pb, were observed. These variations Amp 1–4 were: − 11%, − 9% and − 9% (R ear, L ear and binaural, respectively). The Amp 2–4 Hz parameter in subgroup with higher lead in the blood level showed negative values. The obtained results of the Amp parameter, which describes the emission amplitude, confirm previous research that the damage mainly concerns high frequencies. The result obtained in this paper is consistent with previous studies [13].

The CEOAE signal-to-noise ratio (SNR) is an indicator parameter that eliminates external interference conditions. The SNR excludes external conditions that have a negative impact during the test: e.g. noisy measurement environment, body noises (loud breathing, difficult breathing due to deviated nasal septum, etc.). In this paper, the SNR shows in almost all variants a statistically significant difference between L-Pb and H-Pb, in the results from the R ear, L ear and binaural.

Table 4 presents significant correlations between CEOAE results and years of work exposed to Pb, years of work without Pb exposure. The overwhelming majority of results show a stronger negative correlation with years of work in lead exposure (except: Amp 1–4 on R ear, L ear and binaural, where a stronger correlation applies to years of work without exposure). Years of work exposure also show a positive correlation with PbB and ZPP concentration, averaged over twelve years. This indicates the important role of cumulative exposure to lead and its impact on the sense of hearing. However, the negative correlation of the results of CEOAE parameters and years of work without exposure to lead compounds, and the negative correlation with age, indicates the presence of components of aging of the human body. These results are consistent with data from the scientific literature: the overall response level of CEOAE and the overall reproducibility and amplitude are lower in elderly than in young adults [14]. CEOAE responses are lower in older people, and older people with diagnosed hearing impairment have more reduced CEOAE than people of comparable age with normal (age-appropriate) hearing [15]. The above confirms the correctly selected research methodology, because due to the use of otoacoustic emissions, it is possible to more accurately determine the condition of the external hair cell system. Decrease of overall response level and the overall wave reproducibility may not be separated from the increase in PTA with age, even if PTA remains within the clinically normal range [14].

Many studies have shown a positive correlation between the PbB and Zinc protoporphyrin concentration, ZPP is the lead-dependent biomarker [16]. In this study, we found a higher concentration of ZPP (by 68%) in the group with high lead content, which is consistent with the world literature data.

In this study, we noticed that the cadmium concentration in the blood of workers exposed to lead compounds showed a statistically significant positive correlation with the years of work (in heavy metals exposure), and with PbB and ZPP concentration. Also, the cadmium concentration showed a negative significant correlation with CEOAE results in Amp and SNR parameters, left ear and binaural, mainly at 2–4 Hz. The above research results suggest that exposure to heavy metal mixtures (including Pb and Cd) may cause hearing dysfunction. Most of the studies on the adverse effects on human hearing concerned the population of people exposed to cadmium and lead simultaneously, where hearing loss was positively associated with blood levels of Pb and Cd [17].

The protective effect of calcium has an important role in exposure to heavy metals, the competition of Ca and Pb ions is a factor that modifies the assimilation of this metal, affects the oxidative stress induced by lead, including heme synthesis, and may modify the concentration of cadmium in the blood of workers occupationally exposed to Pb and Cd [18]. In our study, we found that in the H-Pb subgroup the Ca concentration was significantly lower than in the L-Pb subgroup (by 2%). A necessary ingredient for eliciting an action potential of the hair cells is an increase in intracellular calcium concentration, so calcium is necessary to elicit a correct auditory response [19]. Lower serum calcium values may result in more pronounced negative effects of lead on the sense of hearing.

In our work, Zn does not show a statistically significant difference in the subgroups with low and high lead concentration. However, we noticed that there are differences in CEOAE results: zinc concentration correlates statistically significantly positively with CEOAE parameters of the R ear and binaural at frequencies of 2–4 Hz. In world literature there are papers showing that elevated lead concentration is associated with decreased zinc content [20]. Works by other authors also emphasize the protective role of Zn: high zinc level decrease the absorption of lead [21]. On the other hand, single studies report a positive correlation between the content of lead in the blood and zinc in the serum [22]. Opposing results obtained by various authors show the need to conduct further research in this direction.

The sensory cells from the cochlea are very susceptible to oxidative stress caused by both chemical and physical factors. ROS produced as a result of oxidative stress, interacting with the membrane phospholipids of sensory hair cells, form aldehyde products of lipid peroxidation, may lead to hair cells degeneration and apoptosis [23]. In this study, CEOAE results correlated statistically significantly negatively with the MDA concentration. This applies to all tested parameters (Rep, Amp, SNR), in two frequency ranges, ear R, L and binaural. This indicates a possible impact of the content of this lipid peroxidation marker with deteriorating results of CEOAE. In addition, the MDA concentration correlates positively with the cumulative, twelve-year average, blood lead concentration. However, there is a lack of studies on otoacoustic emission in humans with excessive exposure to ROS, let alone oxidative stress caused by occupational exposure to lead compounds. PTA studies in textile workers suggest that there may be a relationship between increased MDA concentration and hearing loss [24]. End products of lipid peroxidation, including MDA, are indicators of oxidative stress, occurring in hair cells, support cells, spiral ganglion neurons and stria vascularis, and can be used in the diagnosis of cochlear function [25].

In our study, LPS concentration was significantly higher in the H-Pb subgroup and shows a negative correlation with otoemission results in the L ear (and with the twelve-year average ZPP value). Autophagy, together with the ubiquitin–proteasome system, are the main degradation pathways—thus abnormal cytoplasmic proteins and organelles are removed intracellularly. The accumulation of LPS in the cochlear cells occurs on the way to increased autophagy and the deposition of this substance in the cochlear cell [26]. Under oxidative stress, intracellular autophagy is impaired, LPS accumulation occurs, which can lead to premature aging of the hair cells [27].

## Conclusions

Long-term higher blood lead concentrations were associated with hearing damage—higher PbB affected the poorer otoemission parameters. The presence of cadmium in the exposure mixture may enhance the negative ototoxic effect of lead. Ca and Zn may have a protective effect of hearing sense.

Long-term exposure to lead causes an increase in the concentration of malondialdehyde, which correlates negatively with all CEOAE parameters tested. This suggests an important role of oxidative stress in the long-term deterioration of hearing. Excessive accumulation of lipofuscin, under conditions of increased oxidative stress, leads to negative changes in hearing function.

## Data Availability

The datasets generated during the current study are available from the corresponding author on reasonable request.
